# Clozapine and risperidone influence on cortisol and estradiol levels in male patients with schizophrenia

**Published:** 2015

**Authors:** G Piriu, E Torac, LE Gaman, L Iosif, IC Tivig, C Delia, M Gilca, I Stoian, V Atanasiu

**Affiliations:** * ”Carol Davila” University of Medicine and Pharmacy, Bucharest, Romania; ** Sapoca Neuropsychiatry Hospital, Buzau, Romania; *** R & D Irist Labmed, Bucharest, Romania; **** ”Prof. Dr. A. Rusescu” Institute for Mother and Child, Bucharest, Romania

**Keywords:** atypical antipsychotics, estrogen, cortisol, men

## Abstract

Estrogens role in schizophrenia patients is a subject, which has gained an increased attention from the medical community. Estrogens have been shown to inhibit dopamine actions, improve neuronal regeneration, and overall, have a protective role in the pathology of schizophrenia. The adjunctive estrogen therapy for men is currently under debate.

Antipsychotic medication is known to influence the hypothalamo-hypophyseal – gonadal axis by inducing variable degrees of hyperprolactinemia. Several studies have found that some of the atypical antipsychotics lower cortisol levels in patients and also in healthy controls.

We have investigated the effects of clozapine and risperidone on estradiol levels in men with schizophrenia. We have also evaluated the levels of prolactin and cortisol, taking into account the possible influence of antipsychotic drugs on both these hormones. Both prolactin and cortisol also have the potential to regulate sexual hormones biosynthesis.

Our study found decreased estradiol levels in men with schizophrenia treated with clozapine and risperidone, while prolactin levels were increased only in the risperidone treated group. Cortisol levels are not statistically significant different between groups.

## Introduction

Schizophrenia is a complex disease having multiple negative impacts on the patients’ life. Clozapine and risperidone are among the first drugs from the so-called “atypical antipsychotics” class used in the treatment of schizophrenia. Atypical antipsychotics are generating their clinical effects without having parkinsonian side effects similar as magnitude with classical neuroleptics [**1**]. Beside their benefits, atypical antipsychotics are far from having any side effects. Metabolic dysfunctions, like increased prevalence of insulin resistance, impaired glucose and lipid metabolism are documented in schizophrenia patients and can further be aggravated by the use of antipsychotics [**2**–**4**]. Hypothalamo-pituitary axis function is also disturbed in schizophrenia patients, elevated cortisol levels being found in drug naive, first psychotic episode patients [**5**–**7**]. Several studies have found that some of the atypical antipsychotics are lowering cortisol levels in patients and also in healthy controls [**8**].

Antipsychotic medication is also known to influence the hypothalamo-hypophyseal axis by inducing variable degrees of hyperprolactinemia, explaining the sexual dysfunction observed in schizophrenic patients [**9**,**10**].

The estrogens’ role in schizophrenia patients is a subject, which has gained an increased attention from the medical community. Estrogens have been shown to inhibit dopamine actions, improve neuronal regeneration and overall, have a protective role in the pathology of schizophrenia [**11**,**12**]. Adjunctive estrogen therapy for men is currently under debate [**13**].

Therefore, we considered it of interest to study the impact of two atypical antipsychotics on estrogen levels in men. We investigated the effects of clozapine and risperidone on estradiol levels in men with schizophrenia. We also evaluated the levels of prolactin and cortisol, taking into account the possible influence of antipsychotic drugs on both these hormones. Both prolactin and cortisol also have the potential to regulate sexual hormones biosynthesis.

## Material and methods

**Subjects**

The subjects selected for this cross-sectional observational study were outpatients treated at a psychiatric hospital in Sapoca, Buzău, Romania. Patients selection criteria were: fit the Diagnostic and Statistical Manual of Mental Disorders IV (DSM-IV) criteria of schizophrenia, a minimum of 3 years’ duration of disease, and a 1-year minimum duration of antipsychotic treatment with clozapine or risperidone. The exclusion criteria were acute or chronic illnesses known to affect the immune, endocrine, metabolic systems, any additional chronic medications. ItemGroup Checklist section of the Schedules for Clinical Assessment in Neuropsychiatry (SCAN) was used in order to confirm the diagnosis.

The study group consisted of 64 patients, all males, European Caucasian, with ages between 24–45; 19 subjects were under risperidone treatment, 30 under clozapine treatment and 15 healthy volunteers used as control group. Risperidone dosages were between 2 and 6 mg daily, while clozapine doses ranged from 100 to 450 mg daily throughout the treatment duration.

The study was approved by the local ethics committee and conducted according to the ethical obligations of the Declaration of Helsinki. All the subjects were provided with a complete description of the study and provided an informed consent to participate in the study.

**Methods**

Estradiol and prolactin were determined by using ELISA kits from DRG International Inc, 841 Mountain Ave, Springfield Township, USA; 17-HO-progesterone and cortisol were determined with ELISA kits from IBL-Hamburg GmbH, 22335 Hamburg, Germany.

**Statistical analysis**

Data analysis was performed by using InStat GraphPad from Graph Pad Software, Inc., La Jolla, CA, USA. Differences between groups were calculated by using a variance analysis (ANOVA) with parametric (Tukey’s) or nonparametric (Kruskal-Wallis) post hoc test. Strength of association was established by Pearson correlation and a P<0.05 was considered statistically significant. 

## Results

Results are presented in **Table 1**. There were no significant statistical differences in cortisol levels between clozapine and control groups (322.07±106.69 ng/ mL vs. 325.41±144.16 ng/ mL). Risperidone group cortisol levels were slightly higher, but also without a statistical significance (395.41±81.32 ng/ mL).

**Table 1:** Hormone profile table. Values are expressed as mean + standard deviation (SD) (Cl=clozapine, R=risperidone, C=control).

	Control	Clozapine	Risperidone	Statistical Significance
n	15	30	19	
Cortisol (ng/ mL)	325.41±144.16	322.07±106.69	395.41±81.323	Ns.
Prolactin (ng/ mL)	9.91±5.06	14.38±13.40	36.35±19.53	C/R ***P<0.001 Cl/R ***P<0.001
Estradiol (pg/ mL)	58.56±24.88	37.46±16.93	37.55±16.94	C/Cl ** P<0.01 C/R *P<0.05
17-OH-Progesterone (ng/ mL)	0.30±0.49	0.88±0.49	1.18±0.77	C/Cl **P<0.01 C/R *P<0.001

Risperidone group prolactin levels (36±19.53 ng/ mL) were significantly higher than those of control and clozapine group, p<0.001. Prolactin levels were slightly higher for clozapine group when compared to control group (14.38±13.40 ng/ mL vs. 9.91±5.06 ng/ mL). There was no significant statistical difference in prolactin levels between the clozapine and control group.

There were no differences in estradiol levels between clozapine and risperidone treated groups (37.46±16.93 pg/ mL vs. 37.55±16.94 pg/ mL). Both showed significantly lower estradiol levels when compared to control group, (58.56±24.88 pg/ mL), p<0.01 clozapine vs. control and p<0.05 for risperidone treated group vs. control.

Risperidone treaded group 17-hydroxyprogesterone levels were significantly higher when compared with the control group (1.18±0.77 ng/ mL vs. 0.30±0.49 ng/ mL) p<0.001, and slightly higher when compared to clozapine treated group levels, without any statistical significance. Clozapine treated group levels (0.88±0.49 ng/ mL), were moderately higher, with p<0.01 when compared to the risperidone group.

A significant positive correlation between 17OH-progesterone and cortisol (p=0.0123, r=0.458) was found in clozapine. All the other correlations investigated for Control and Risperidone groups showed not significant results.

## Discussion

As expected, prolactin levels in the risperidone treated group was significantly higher than prolactin levels in clozapine treated group and then in controls. These results were in agreement with the previously research data found in literature [**14**,**15**]. No statistically significant clozapine influence on prolactin levels was noticed when compared with the controls.

Interestingly, in both groups of patients, clozapine and risperidone treatment were decreasing estradiol levels. 

In men, estradiol production is mainly done in adipose tissue, brain, adrenals, liver and gonads [**16**]. Estradiol synthesis from testosterone is done by aromatase. The level of estradiol in men is therefore dependent on testosterone concentration and on aromatase activity.

Testosterone concentration is under the control of the hypothalamo-hypophyseal axis. While risperidone is clearly affecting this ax by increasing the level of prolactin we cannot say the same about clozapine whose effect on prolactin level was not significant in our study. However, previous studies on risperidone effects on the hypothalamo-hypophyseal ax have shown no inhibitory influence of higher prolactin levels on the control of testosterone synthesis [**10**].

Aromatase is an enzyme belonging to cyt P450 family. Cortisol is proved to increase the activity of aromatase in adipose tissue [**17**]. Interestingly, this effect is observed only in women and not in men. However, we did not find statistically significant differences in cortisol levels between patients groups and the controls in our study. 

Taking into account that both clozapine and risperidone are metabolized by cyt P450, even by different subfamilies, it might be possible that both drugs are affecting aromatase activity.

## Conclusion

Our study has found decreased estradiol levels in men with schizophrenia treated with clozapine and risperidone, while prolactin levels were increased only in risperidone treated group. Cortisol levels are not statistically significant different between groups.

**Acknowledgement**

Gabriela Piriu and Elena Torac equally contributed to this work as the first authors. Ms. Elena Torac was supported by the doctoral program POSDRU/159/1.5/S/137390, from the European Social Fund.

**Conflict of interest**

All the authors have nothing to disclose.
